# Synergistic effect of land-use and vegetation greenness on vulture nestling body condition in arid ecosystems

**DOI:** 10.1038/s41598-018-31344-2

**Published:** 2018-08-29

**Authors:** Andrea Santangeli, Orr Spiegel, Peter Bridgeford, Marco Girardello

**Affiliations:** 1 The Helsinki Lab of Ornithology, Finnish Museum of Natural History, University of Helsinki, P.O. Box 17, Helsinki, FI-00014 Finland; 2 School of Zoology, Faculty of Life Sciences, Tel Aviv University, Tel Aviv, 69978 Israel; 3 Vultures Namibia, Walvis Bay, Namibia; 4 cE3c - Centre for Ecology, Evolution and Environmental Changes/Azorean Biodiversity Group and Universidade. dos Açores – Depto de Ciências e Engenharia do Ambiente, PT-9700-042 Angra do Heroísmo, Açores Portugal

## Abstract

Climate-driven environmental change and land-use change often interact in their impact on biodiversity, but these interactions have received little scientific attention. Here we study the effects of climate-driven environmental variation (i.e. vegetation greenness) and land-use (protected versus unprotected areas) on body condition of vulture nestlings in savannah landscapes. We combine ringing data on nestling measurements of two vultures (lappet-faced and African white-backed vulture) with land-use and environmental variables. We show that body condition of white-backed vulture nestlings decreased through the study period and was lowest inside protected areas. For the lappet-faced vulture, nestling condition was improved during harsh years with lower than average vegetation greenness assumed to result in increased ungulate mortality, but only within protected areas. Such interaction was not tested for the white-backed vulture due to collinearity. The species-specific effects of land-use and vegetation greenness on nestling condition of the two sympatric vulture species likely stem from their different life-histories, diet preferences and foraging behaviour. While translation of current findings on nestling conditions to their possible influence on population demography and species persistence require further studies, our findings demonstrate how environmental change may trigger selective bottom-up ecosystem responses in arid environments under global change.

## Introduction

Climate change and land-use change are among the major drivers of biodiversity loss^1,2^. Impacts of climate change are predicted to be particularly important in already fragile areas with low ecosystem resilience^3^. Anthropogenic land conversion and climate change may often act in synergy, triggering a negative feedback whereby habitats may rapidly deteriorate, resulting in wildlife population declines and possible extinctions^4^. Such synergistic impacts may be particularly important in most regions of the world where large swaths of land are under some form of production regimes (such as farmland) and are at high vulnerability from climate change^3,5^.

Among those regions under rapid anthropogenic conversion and of high vulnerability to climate change are the arid savannahs of Africa^5^. This biome was recently found as highly endangered, as most of it is currently unprotected^6^. The arid savannahs of Africa support important taxonomic and phylogenetic diversity^7^, provide key ecosystem services, such as food and fiber, and generate profit from ecotourism that contribute to improve local livelihoods^8^. These areas, once largely natural, are now being widely converted to farmland, either for livestock or for crop production^9^. In these man-managed arid savannah landscapes, relatively small shifts towards harsh conditions triggered by climate change may strongly impact on the life-history of some species^10–13^.

Land-use and environmental change may have particularly disproportionate impacts on specialist species at the top of the food chain, such as obligate scavengers which rely on multiple trophic levels for their survival and reproduction^14^. As the sole obligate scavengers, vultures play an important role in the savannah ecosystem, and across many other terrestrial systems of the world^15^. By comprising an exclusive functional guild, vultures contribute to clean the environment from decomposing organic matter and keep the ecosystem in balance^15,16^. Carrion, the primary food source for vultures, constitutes a largely unpredictable and localized resource that is also used by competitor facultative scavengers^14,15^.

While anthropogenic activities are causing widespread vulture declines^15,16^, in the absence of anthropogenic-driven mortality, vulture populations are generally limited by resource availability and their slow reproductive rates^14^ which are typical of large-bodied long-lived species often representing K life history strategy^17^. The ranging behavior and distribution of vultures are strongly related to the availability of carrion^18–21^. Ultimately, food availability for vultures will be determined by the abundance of wild ungulates (or domestic livestock) and their mortality rate, which may vary in space and time in response to environmental conditions and management practices^22^. Carrion availability will also depend on the presence and abundance of mammalian competitors, as well as carcass visibility and accessibility^23,24^.

Environmental conditions may directly affect the body condition of nestlings (e.g., through thermoregulation and water stress^11^) or indirectly through their influence on foraging success and movement patterns of their parents^25^. Nestling body condition may ultimately determine breeding success or survival during the post-fledging period and consequent recruitment to the breeding population^26^. For example, environmental conditions experienced early in life were found to affect future survival prospects of long-lived vulture species^27^. There is a large body of scientific work on the movement and distribution of vultures in relation to the coverage of protected areas as well as spatio-temporal variation in environmental conditions^19,22,28,29^. However, studies on the effects of environmental and climate-driven drivers on the breeding biology of obligate scavengers are still relatively scarce. More generally, studies addressing the interactive impacts of land-use and environmental change on species life-histories are still very rare, hindering the development and implementation of appropriate adaptation measures under global and local changes^30^.

**Figure 1:**
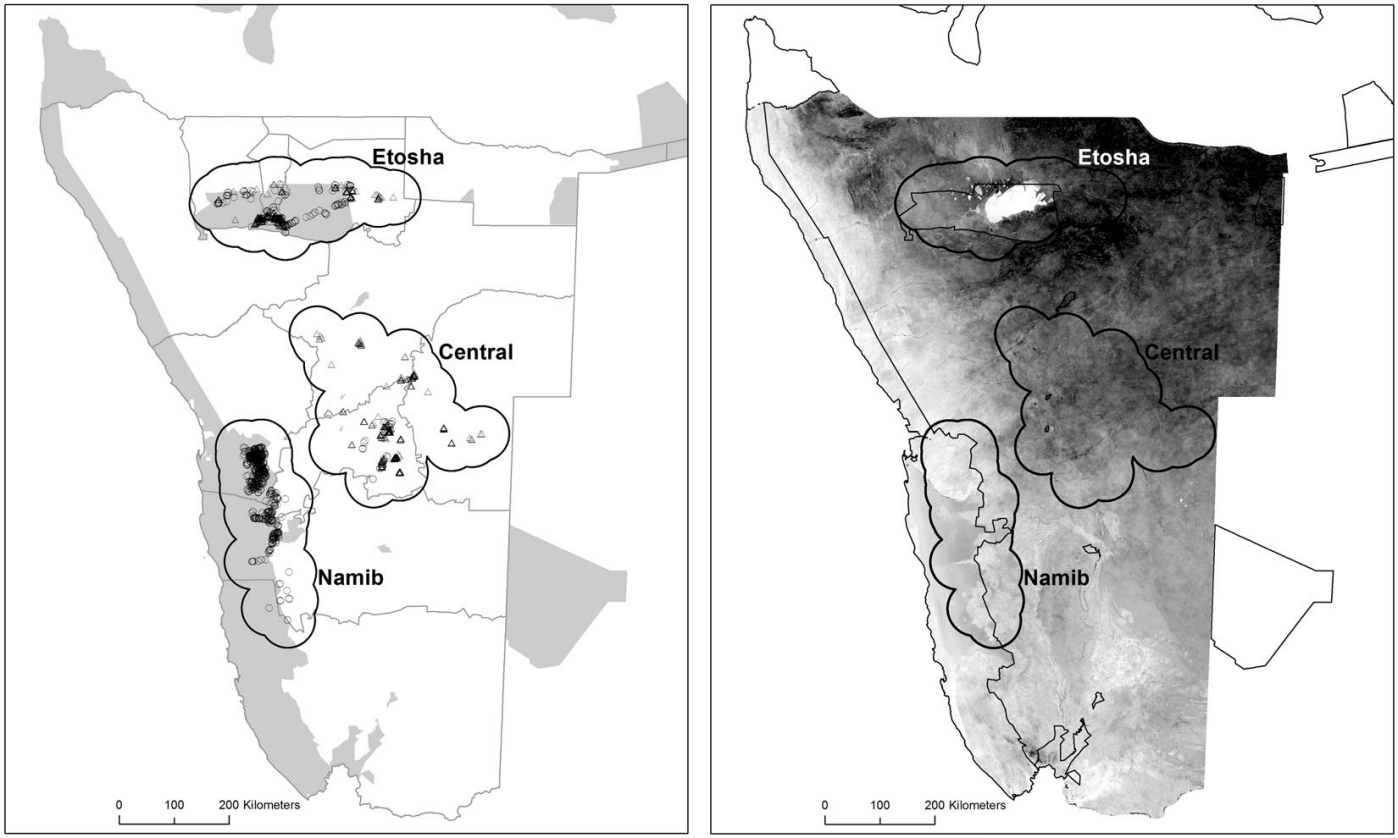
Map of Namibia with the location of the three study regions (delimited with thick black line). Left panel shows also the cover of protected areas (grey shades) and the locations of nest where nestling measurements for the lappet-faced (empty circles) and white-backed (empty triangles) vultures included in this study. The right panel shows the normalized difference vegetation index (NDVI) across the study landscape, in this case representing the situation as an yearly average NDVI value for the year 2007. Figure created in ArcGIS 10.1.0 software (http://desktop.arcgis.com/en/). The polygons of administrative borders and protected areas of Namibia were retrieved from http://www.nnf.org.na/eis/30.html a free online information resource. The NDVI dataset used was acquired from NASA LP DAAC. MODIS/Terra + Aqua Vegetation Indices 16-Day L3 Global 250 m (MOD13Q1). USGS Earth Resources Observation and Science (EROS) Center, Sioux Falls, South Dakota (https://lpdaac.usgs.gov), accessed January 31, 2017.

In this study we explore the independent and interactive impact of extrinsic factors on nestling body condition of two obligate scavenger species, the lappet-faced (*Torgos tracheliotus*) and African white-backed (*Gyps africanus*; hereafter white-backed vulture) vulture, in an arid ecosystem of Africa. For the white-backed vulture, only independent impacts were explored due to collinearity (see methods). We hypothesize that forage availability for ungulates (here quantified using a measure of vegetation greenness) could affect ungulate mortality (e.g. during prolonged periods of poor forage availability) and thus carcass availability for vultures, in turn affecting nestling condition. We predict that harsh, dry conditions assumed to increase mortality rates of ungulates will benefit carrion eating vultures. We used a unique dataset of ringing observations collected on the populations of these two species in Namibia (Fig. 1). The area experiences rather extreme environmental conditions, e.g. prolonged and intense droughts. The large dataset, accumulated over 14 years and across protected and unprotected areas provides a unique spatio-temporal coverage for testing our hypothesis. Specifically, we aim to quantify how nestling body condition is affected by (i) the cover of protected areas around a nest and (ii) environmental conditions (i.e. climate-driven forage availability for ungulates). The spatio-temporal extent of the dataset also allows us to test (iii) the sensitivity of the results to different temporal scales of changes in environmental conditions (i.e. short or prolonged periods of below-average forage availability assumed to affect ungulate mortality, and in turn nestling body condition). Protected and unprotected areas represent contrasting environments which may be associated with different ungulate and livestock mortality rates under stressful environmental conditions. For example, dry periods may reduce water and forage availability for ungulates in protected areas, resulting in higher mortality there as compared to unprotected farmland areas where constant water and forage are provided to livestock. Therefore, we also tested (iv) for interactive effects of climate-driven environmental conditions and protected areas on nestling body condition.

## Results

**Figure 2:**
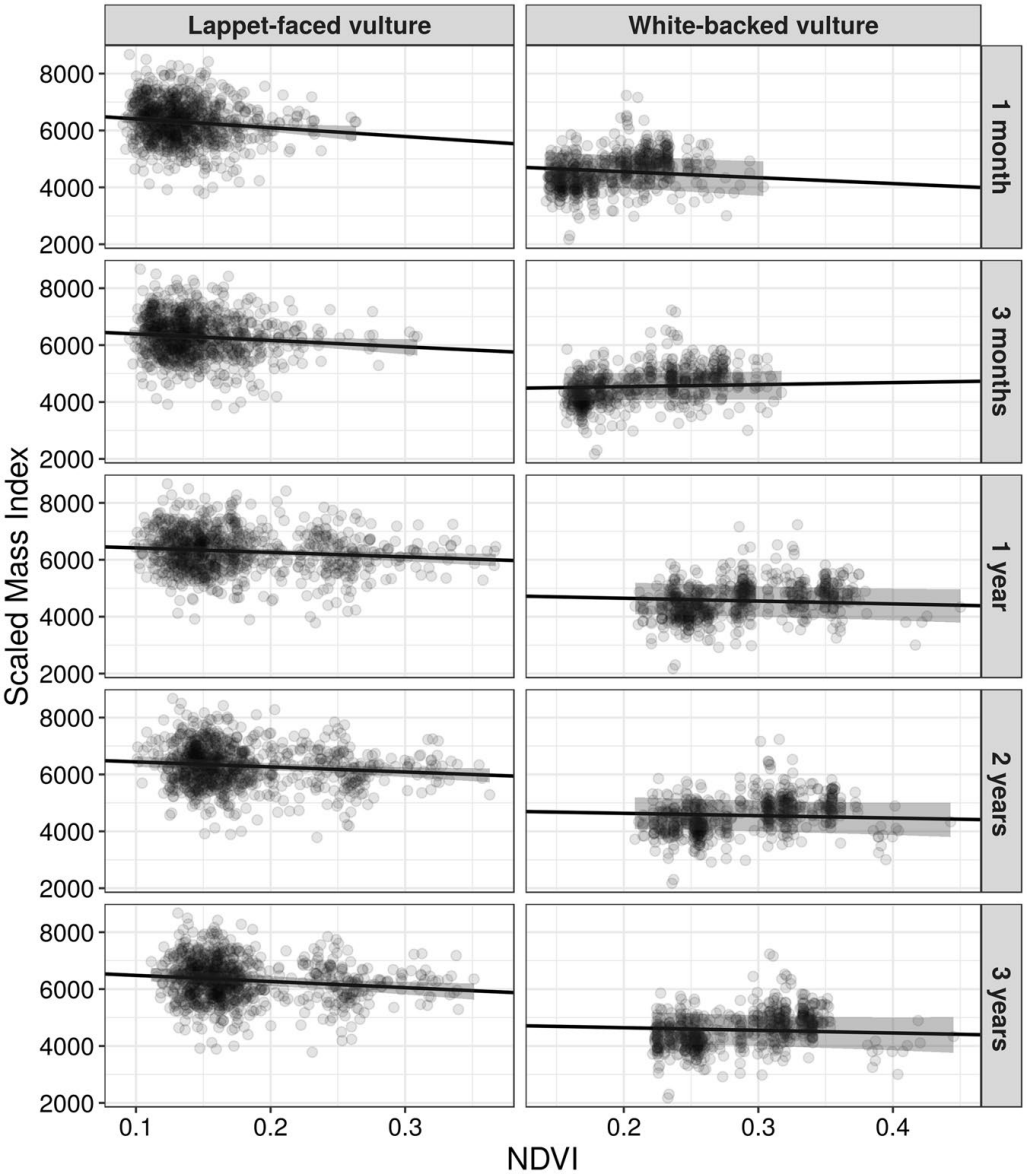
The relationship between the body condition of lappet-faced (left panels) and white-backed (right panels) vulture nestlings and the normalized difference vegetation index (NDVI). We explored NDVI effects across five temporal scales representing the situation during the one month prior to nestling measurement (uppermost row) and up until 3 years prior to the measurement (lowermost row). Each panel shows the raw data (circles) and the fitted linear relationship obtained from the Bayesian hierarchical models (straight black line, with 95% credibility intervals shown as grey shade; see methods and Table 1).

**Figure 3:**
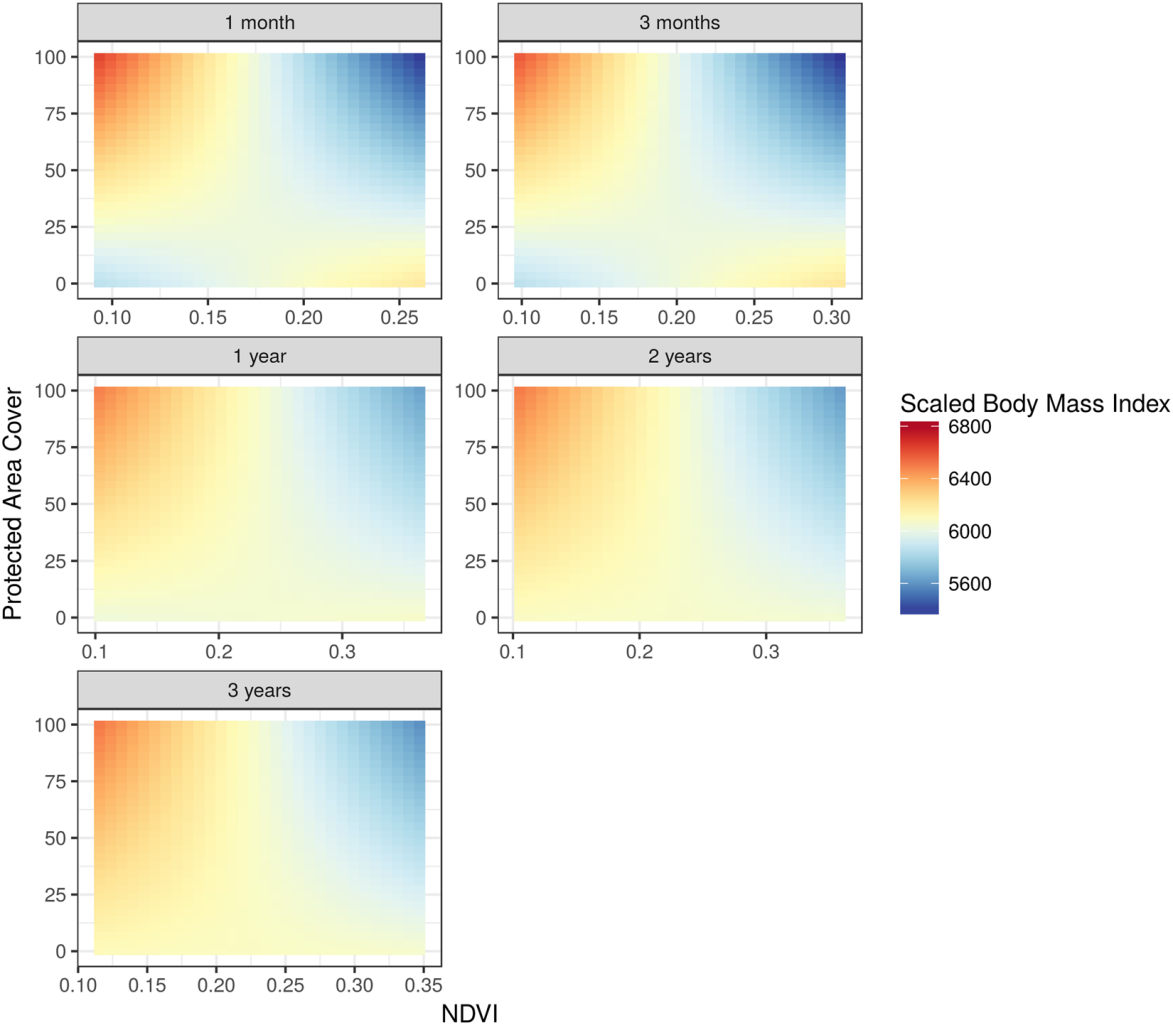
Predicted body condition (here in the form of the scaled body mass index) of lappet-faced vulture nestlings in relation to the normalized difference vegetation index (NDVI) and the cover of protected areas around the nest. The different panels show results obtained by using NDVI calculated at five temporal scales - representing the situation during the one month prior to the measurement and up until 3 years prior to the measurement. These predictions are derived from the model including the interaction term between protected area cover and each of the five NDVI measures (see methods for further details). Warm colors indicate better condition compared to cold colors.

Body condition of lappet-faced vulture nestlings was negatively related to NDVI (Table 1 and Fig. 2). This implies that body condition of lappet-faced vulture nestlings was higher when environmental conditions prior to nestling measurement were drier (lower vegetation greenness, or productivity). This pattern was not sensitive to the period considered for calculating the NDVI, as it was similar for all five temporal scales (from 1 to 36 months; see Table 1 and Fig. 2). Moreover, we found an interactive effect of the protected area coverage and NDVI on body condition (Table 1). This effect was strongest, i.e. the upper and lower credible intervals of the posterior mean did not overlap zero, for the NDVI referring to the shortest periods (one and three months) preceding the body condition measurements (Fig. 3, Table 1 and Fig. S2). Specifically, the effect of protected area on nestling body condition was most positive after dry periods, i.e. low NDVI during preceding months, but became negative after wet periods (Fig. 3). Body condition of white-backed vulture nestlings decreased with the cover of protected areas around the nest, but was not affected by NDVI at any of the five temporal scales considered (Table 1, Figs 2 and S3). Moreover, white-backed vulture nestling body condition declined markedly over the 14 years of study (Table 1 and Fig. S3). A similar but weaker decline in condition seems also apparent for lappet-faced vulture nestlings.

**Table 1:** Summary of the posterior marginal distributions for Bayesian hierarchical models exploring the relationship between the body condition of lappet-faced and white-backed vulture nestlings in relation to the proportion of area protected (PA cover), and the normalized difference vegetation index (NDVI).

Variable	Lappet-faced vulture			White-backed vulture		
	Mean	Lower CI	Upper CI	Mean	Lower CI	Upper CI
PA cover	0.07	−0.01	0.15	**−0.39**	**−0.70**	**−0.07**
NDVI 1 m	**−0.12**	**−0.20**	**−0.03**	−0.08	−0.22	0.07
NDVI 3 m	**−0.10**	**−0.18**	**−0.01**	0.13	−0.03	0.29
NDVI 12 m	**−0.14**	**−0.23**	**−0.05**	−0.01	−0.14	0.13
NDVI 24 m	**−0.14**	**−0.23**	**−0.05**	0.06	−0.12	0.23
NDVI 36 m	**−0.15**	**−0.25**	**−0.06**	0.14	−0.07	0.34
Time	−0.07	−0.14	0.00	**−0.16**	**−0.26**	**−0.07**
NDVI 1 m * PA cover	**−0.06**	**−0.11**	**−0.01**			
NDVI 3 m * PA cover	**−0.06**	**−0.10**	**−0.01**			
NDVI 12 m * PA cover	−0.04	−0.09	0.01			
NDVI 24 m * PA cover	−0.04	−0.09	0.01			
NDVI 36 m * PA cover	−0.04	−0.09	0.01			

We calculated NDVI at five temporal scales representing the situation during the one month prior to the measurement (NDVI 1 m) and up until 3 years prior to the measurement (NDVI 36 m) and modeled these separately. PA cover and the NDVI refer to the area within a 50 km radius around the nest (see methods). The variable named time aimed to depict continuous inter-annual trends in body condition. Each variable and related statistics are derived from separate models including only that variable and time as continuous in the fixed part of a model (see methods and Appendix S1). The statistics for the main variables effects were derived from models without interactions. Values represent the mean of the posterior distribution with upper and lower 95% credibility intervals. Variables for which the credibility intervals do not overlap zero are shown in bold font.

## Discussion

We show that the effects of land-use, i.e. coverage of protected areas, and climate-driven conditions, i.e. NDVI, on vulture nestling body condition are species-specific and can be interactive. Partly in accordance with our initial hypothesis, we found that, for lappet-faced vulture nestlings only, body condition is increased after periods of low availability of forage for ungulates (below average NDVI, presumably linked to increased ungulate mortality), and that this effect is mediated by land-use, i.e. relative coverage of protected areas. Specifically, at nests largely surrounded by protected areas, body condition increased after periods of low availability of ungulate forage, but body condition worsened following periods of high forage availability. White-backed vulture nestlings, however, were not influenced by variation in forage availability, their body condition was lower at largely protected nests and also declined through the study period.

Body condition of altricial nestlings is typically mediated by the amount and quality of food that the parents can provide^17^. The finding that white-backed vulture nestlings are in better condition in unprotected as compared to protected areas is interesting. Unprotected areas in Namibia are largely dominated by livestock and game farms. As obligate scavengers, vultures may rely on livestock mortality as well as ungulates killed by predators and for trophy hunting at game farms, a growing industry in Namibia’s unprotected land^31^. As white-backed vultures are social foragers that roam large areas in search of food^14,20^, they may more readily take advantage of the carrion availability offered by livestock and game farmland as compared to the lappet-faced vultures that typically forage in pairs^20^. These contrasting foraging strategies may likely contribute to explain the result reported above. This assertion is also in line with previous findings from South Africa suggesting that white-backed vultures can readily take advantage of carrion provided by expanding game and cattle farms^32^.

Climatic conditions were found to be important determinants of nestling body condition and breeding success in a number of species^11,33,34^, with reported effects of climate being direct, e.g. causing physiological stress^11^, or indirect, causing changes in food availability^34,35^. Ungulate populations of the African savannah are strongly regulated by primary productivity, which is mediated by climatic factors, such as rainfall, and in turn impacts on the food base for vultures, with repercussions on their movement, reproduction and survival (see e.g.^36^). In arid savannah landscapes, such as those of most of Namibia, below average rainfall likely triggers additional ungulate mortality, due to reduced forage availability^22,37,38^. In this study, NDVI was used as a proxy for ungulate forage availability and is presumably inversely correlated with their mortality. The increased ungulate mortality resulting from below average forage availability (i.e. low NDVI) likely leads to increased carrion availability for the lappet-faced vultures and improved nestling condition in those years.

Putting our findings into a broader context of longer time periods, we note that although low ungulate forage availability within protected areas may improve lappet-faced vulture nestling condition in the short-term through the boosted food availability from ungulate mortality, this benefit may be ephemeral. Arid savannah landscapes, such as those of Namibia, are expected to experience heavy droughts under climate change^3,5,39^. In the long run, this may cause desertification, with consequent reduction in the carrying capacity of the environment and overall declines of ungulate numbers^5,40^, ultimately reducing the food base for vultures.

The fact that NDVI (i.e. ungulate forage availability) did not seem to affect white-backed vulture nestling condition is surprising. It is possible that adult white-backed vultures are able to better buffer against climate-driven environmental variability as compared to the lappet-faced vultures. For example, white-backed vultures typically roam across larger foraging areas, which may support adequate food resources during favorable and adverse years, as compared to the lappet-faced vultures^20^. White-backed vultures also rely on larger animals as a food source compared to lappet-faced vultures^14^. Larger ungulates may be more resistant to harsh environmental conditions (e.g. decreased food quality) as compared to smaller ungulates which may be also impacted by increased predation in dry years^40^. Consequently, years of poor forage availability for ungulates may selectively increase small ungulate mortality, and favor the lappet-faced vulture more than the white-backed vulture. Moreover, and perhaps most importantly, the white-backed vultures are social foragers, which enables them to scan larger areas more efficiently as compared to the lappet-faced vultures that typically forage in pairs^14,20^. This feature of the white-backed vultures may allow them to efficiently gather around and take advantage of already discovered carcasses, irrespective of climate-driven yearly variation in carrion abundance. The finding that white-backed vulture nestlings seem to buffer their body condition against varying environmental conditions requires further ad-hoc investigations beyond the scope of this study.

Interestingly, body condition of lappet-faced vulture nestlings was strongly affected by the interaction between protected area cover and climate-driven ungulate forage availability. Nestling body condition was most improved after years of poor forage availability for ungulates but only in largely protected sites. Indeed, the effect of variation in forage availability on nestling body condition within protected areas is rather large, amounting to a gain of approximately one kg of body mass when environmental conditions change from the highest to the lowest forage availability (corresponding to highest to lowest NDVI values in Fig. 3). The gain of one kg described above appears remarkable considering the average weight of just above six kg of the lappet-faced vulture nestlings considered here. We propose that environmental stressors for ungulates may be most pronounced within ecosystems found in protected areas as compared to man-managed unprotected landscapes. In the latter, water and pasture scarcity may be buffered by the food and water supplementation or by artificial reduction in stock density. Thus, while in protected areas lower forage availability may lead to additional available carrion for local lappet-faced vultures (as suggested by the improved nestling condition under low NDVI), this is not the case in farmland dominated unprotected landscapes.

Outside of the protected areas of the world, the human footprint is increasing, causing changes across species, ecosystems and habitats^4^, including space use patterns of various species^41^. Under these changing conditions, our finding that lappet-faced vultures in unprotected land did not benefit from increased resource availability like their counterparts in protected areas highlights an important prediction for future dynamics of this species. Predicted changes in land-use (i.e., farmland expansion and/or changes in farm management practices) accompanied by climate change will further restrict species ability to benefit from harsh conditions and to cope with environmental change. This suggests that while protected areas are very important under current conditions, in the near future they will play an even more crucial role in preserving healthy ecosystems where vultures can benefit from periods of favorable conditions (and unfavorable for ungulates).

In the long term, however, climate change may trigger vulture distribution range shifts, pushing them progressively outside of the current protected area network, as shown by a recent study on the cape vulture (*Gyps coprotheres*)^42^. Therefore, an immediate challenge is to ensure the preservation of healthy ecosystems within the current protected areas and address the threats to vultures in unprotected landscapes dominated by livestock and game farms. Here, the use of poisons to kill carnivores^43,44^ as well as environmental contaminants^45^, such as lead present in hunted game, represent impelling threats that need to be urgently addressed. This would allow for the short as well as long term conservation of vulture populations across arid savannah landscapes under rapid environmental change.

This work represents one of the very few cases (but see^33^) where climate effects, acting in synergy with land-use, are shown to impact nestling body condition, with potential, yet currently unknown, demographic consequences. Juvenile vultures are typically characterized by low survival as compared to adults^46,47^. Nestling body condition was found to have carryover effects on survival of several species^48,49^. Therefore, the improved nestling condition of lappet-faced vultures in protected areas under dry conditions, and the importance of livestock and game farmland for white-backed vulture nestlings, carry important conservation implications. They highlight the need for implementing conservation actions within and outside protected areas to maintain viable vulture populations in the long term. A broad landscape level approach is crucial for species like obligate scavengers that roam very large areas, often well beyond the protected area networks^19^.

The worsening trend in body condition of white-backed vulture nestlings over the 14 years study period may be explained by deteriorating environmental conditions, particularly outside of protected areas. Here, while the recent expansion of the game farm industry may provide an increased food source for vultures, it may also negatively affect their condition through increased exposure to lead from hunting ammunitions^45^. Another factor that may explain the negative trend in body condition is the progressive bush encroachment (i.e. the increase in woody vegetation cover), that may reduce the detectability and accessibility of carcasses to vultures. This growing phenomenon is occurring particularly across Namibia’s livestock farmland^50^ and was indicated as one of the causes of the decline of the national cape vulture population^51^. The fact that the decline in body condition is strongest for the white-backed vulture as compared to lappet-faced vulture nestlings may be due to their different foraging ecology and recent population trends. Populations of these two vulture species have declined over the past decades in the study region^43^, as well as across Africa, largely due to poisoning^52^. These declines in number may have affected the foraging efficiency of white-backed vultures, which are social foragers and rely on social information to detect carrion to a much larger extent than the lappet-faced vultures^20^. The resulting decline in foraging efficiency of the white-backed vultures when its numbers become scarce likely translates in less food provided to the nestling. This may contribute to the decline in body condition observed here. The direct causal link portrayed above would however warrant further ad-hoc investigations.

While we show here that vulture nestlings’ body condition may vary according to the coverage of protected areas and environmental conditions, our correlative study does not allow to establish causation in the patterns found. Nevertheless, these results underscore that the variation in environmental conditions, in combination with land-use, may trigger measurable changes in nestling body condition, as shown for the lappet-faced vulture (Fig. 3). These changes may carry over to the successive stages of the birds’ life, but the consequences are largely unknown. However, we can speculate that an excess of one kg in body mass may make a disproportionate difference for a nestling of a species that largely relies on occasional and unpredictable food, such as carrion. In such extreme conditions, extra body resources may allow a nestling to survive through periods of food scarcity. Thus, our findings can pave the way to future work which could explore the carry-over effects of nestling body condition on survival, recruitment and ultimately fitness, ideally using a semi-natural experimental design. Such investigations would require very long-term and individual-based data given the long lived and slow reproducing features characterizing vultures. They would however be particularly relevant in light of the observed worsening I body condition of the white-backed vulture, an IUCN (International Union for Conservation of Nature; www.iucn.org) Critically Endangered species. Understanding potential demographic consequences of the observed trend in nestling condition will allow informing the conservation management for this threatened species.

Information on nestling sex was also not available in the dataset used here. Sexing vultures would require DNA extraction, because the sexes are phenotypically alike in both studied species^14^. Biased sex ratio at hatching has been found to be related to environmental conditions in broods of species with a clear sexual-size dimorphism, such as the Tengmalm’s owl (*Aegolius funereus*)^53^. Given the lack of sexual-size dimorphism in the two vulture species considered here, we deem unlikely that nestling sex would represent a confounding factor in our analyses.

Climate-driven environmental change and land-use change may act independently but often interact in their impact on biodiversity^30^. Our findings carry timely implications for preserving healthy savannah ecosystems through the adoption of adaptive management strategies that would ensure the preservation of viable vulture populations within and beyond protected areas under global change. Ultimately, this can only be achieved if the drivers of change, and their interactive impacts, are well understood.

Understanding the drivers of change and the mechanisms associated to them is crucial for predicting and managing impacts on biodiversity under global change. Here we provide evidence of species-specific interactive impacts of different drivers of change on the life-history of a critical functional guild^15^. The finding that two important drivers (such as land-use and climate-mediated vegetation productivity) act in synergy in their influence on lappet-faced vulture nestling condition further highlights the importance of considering interactive effects of multiple drivers of change.

## Methods

### Ethics Statement

The work was conducted in accordance with all relevant national and international guidelines. The handling and measuring of nestlings were carried out by experienced bird ringers holding a valid SAFRING (South African Bird Ringing Unit) ringing license approved by the Namibian Ministry of Environment and Tourism, and following the guidelines for ringing provided by SAFRING (http://safring.adu.org.za/).

### Study species and study landscape

The study was based in Namibia, a country dominated by arid savannah landscapes, with low yearly rainfall largely concentrated during the period October to March^54^. While most of Namibia is covered by sparse savannah woodland, the coastal areas form a barren and typically sandy desert, such as that protected within the Namib-Naukluft National Park. In this study we focused on three discrete regions where most of the ringing data of vulture nestlings are available (see Fig. 1). A northern region (hereafter named Etosha), is partly included within the Etosha National Park. A central region (hereafter Central) which lies on the highlands of central Namibia, and is largely represented by commercial livestock farmland. A third region is partly included within the Namib-Naukluft National Park (hereafter named Namib; Fig. 1). Unprotected areas within the study region are typically managed for livestock farming, game farming or a mixture of the two, whereas crop farming is scarce in these regions due to the arid conditions^54^. Part of these unprotected areas belong to commercial farmers, and part are used by communal subsistence farmers which typically belong to conservation conservancies^8^.

We focused on the two most common breeding species of vultures in Namibia, the lappet-faced and the white-backed vulture^55^. The sexes of both species are phenotypically alike and can only be separated by means of DNA analyses^14^. These two species typically nest on trees in the dry savannah, mostly represented by Acacia species^55^. The lappet-faced vulture and the white-backed vulture are classified as Endangered and Critically Endangered by IUCN owing to their rapid population declines that are likely to continue into the future^52^. The main threats to both species are intentional and unintentional poisoning, habitat degradation and collision mortality with infrastructures^43,44,52^. In Southern Africa, breeding occurs during the Austral winter, with hatching taking place during the middle of the dry season, typically between July and August. Both species lay one egg and the nestling period lasts about four months^14^. This period coincides with progressively drying conditions in Namibia, which typically exacerbate towards the second half of the nestling period, between September and December^54,55^. In this way, vultures synchronize the most energy demanding phase of their life cycle with a pulse in food availability resulting from increased ungulate mortality induced by low availability of nutritious vegetation during dry periods^22,36^.

### Nestling measures and calculation of body condition

We use nestling ringing data for the period 2003–2016 when ringing effort was high and constant throughout. Nests have been typically located either through systematic surveys from the ground or from the air (as in the case of Etosha and the Namib regions). Alternatively, nests were located by opportunistic sightings of the nest with adults by the ringer, farmer or farm-workers (as in most of the observations for the Central study area). Nestlings were ringed at the age between two months and before fledging. Ringers typically reached the nest using a ladder. Handling involved placing a metal ring on the leg and measuring the wing length and the weight of the individual chick. The chick was then placed back to the nest, with the whole procedure lasting half an hour on average. Overall, the dataset includes 608 white-backed and 899 lappet-faced vulture complete data points (i.e. including body weight, wing length and coordinates of each observation). Of these, 292 and 316 white-backed vulture data points were available for the Central and Etosha regions, respectively. No ringing data for this species are available for the third region, the Namib, as white-backed vultures did not breed there. For the lappet-faced vulture, 730, 26 and 143 ringing data points were available from the Namib, Central and Etosha regions, respectively.

Body condition was estimated for each nestling from the mass and wing length measures and using the scaled mass index method^56^. This index scales each individual body mass to the expected value if all nestlings had the same body size. The scaled mass index is deemed highly robust to measurement errors and allows reliable comparability of body condition from measurements taken on subjects with different age^56^. It also provides a more reliable indication of the true condition of an animal compared to other approaches, such as the common one based on residuals calculation from an ordinary least square regression of body mass against length^56^. The formula for calculating the scaled mass index used here is as follows: body mass × (464.16/wing length)^0.69^ for the lappet-faced vulture nestlings, and body mass × (410.94/wing length)^0.65^ for the white-backed vulture nestlings. In these formulas, the values within brackets represent the mean wing length of the population of each species (in mm) to which all body mass values are adjusted to, respectively, and the upper case coefficients are the slopes of standardized major axis regressions coefficients (see^56^ for more details). In practice, because the index scales the body mass of each individual to the average body size of the population under study, the scaled mass index can be compared among individuals for which the measurements were taken at different ages. The scaled mass index has been successfully used to estimate body condition of adults and nestlings of several species (see e.g.^57^). For simplicity and clarity, we will use the term body condition instead of scaled mass index throughout.

### Predictors of body condition

In order to test the above mentioned hypotheses we extracted the cover of protected area (hereafter PA; see below) and normalized difference vegetation index (NDVI, here used as a proxy for climate-driven ungulates forage availability; see below) from an area with a circular buffer of 50 km radius around the nest location. The rationale for choosing the 50 km radius was based on known movement ecology of the two study species in Namibia indicating that most of the daily foraging movements occur within this distance^20^. Although this is a somewhat arbitrary spatial threshold, we deem this distance to represent an optimal compromise between type I and II errors given the available data quality. On one hand, it is a large enough radius to likely incorporate most of the foraging area of the two species during the breeding season (presumably minimizing omission of utilized area, i.e. Type I error). On the other hand, it is restricted enough so that it includes only areas that are directly relevant for foraging, avoiding areas not utilized by the feeding parents (thereby confounding and possibly masking some genuine patterns, i.e. Type II error). We found very strong spatial autocorrelation in the NDVI data (see Fig. 1 right panel) and thus we argue that radii of different sizes would most likely lead to very similar results as those obtained with the current selection.

Protected areas were extracted as polygon files for GIS software from the World Database on Protected Areas (WDPA; https://protectedplanet.net/). We selected only areas belonging to the IUCN protected area categories from I to IV that are currently designated (largely represented by National Parks, see Fig. 1 left panel). PA cover was calculated as the percentage area protected within the 50 km buffer around each nest. Due to restrictions on resource extraction (including hunting) and human land-use (e.g. farming), protected areas typically support ecosystems at a more “natural” state as compared to unprotected sites. In PAs wild ungulate populations are at the mercy of the environmental variation (e.g. prolonged dry periods; although localized water points may exist in some of the PAs) and carcasses are left *in situ*, thus providing food for vultures, as compared to unprotected land (largely dominated by livestock farmland where water provision is regular) where dead animals may be removed^32,54^. Thus, protected areas may positively affect the body condition of vulture nestlings.

In addition, we also calculated the mean NDVI value for the same 50 km buffer area described above. NDVI offers a remotely sensed measure of the greenness of the vegetation, which is in turn related to primary productivity and forage availability for wild and domestic ungulates^22,42,58^. NDVI has also been related to ungulate population demography, including increased mortality under prolonged droughts^59,60^. This may also apply to domestic livestock in Namibia^44^. Therefore, we calculated NDVI for five different time periods, spanning backwards from nestling measurement date for one month, three months, one, two and three years. This temporal gradient in the lag of NDVI effects allowed to explore whether prolonged periods of below average NDVI values, which means prolonged drought, positively correlate with vulture nestling condition through increased wild and domestic ungulate mortality. The original NDVI values used in this study were available at 16-day composite values with a resolution of 250 m X 250 m and obtained from^61^.

### Statistical analyses

We used Bayesian hierarchical models to examine the relationship between nestling body condition and PA coverage and the five NDVI timescales, all as continuous variables. Year was included as a continuous covariate in all models (in this case it was named *time* for clarity, see Table 1) in order to account for unexplained temporal trends in the data. Region identity was included as random factor (Etosha, Central and Namib). This random structure was defined a priori to account for pseudo-replication due to potential measurements of nestlings from the same pair within the same region. To deal with the potential confounding effects of spatial autocorrelation, we also included in the models a spatially correlated random intercept term. The random intercept is assumed to be a Gaussian Markov Random Field (GMRF) with mean 0 and covariance matrix ∑. Because of the difficulty in estimating a covariance matrix for large datasets, an approach based on Continuous Domain Stochastic Partial Differential Equations (SPDE) is used to calculate the covariance matrix ∑ for the GMRF^62^. This is a computationally efficient approach for large datasets, as it avoids the computation of a full covariance matrix for the GMRF. The models were fitted using integrated nested Laplace approximations (INLA). INLA was chosen as it allows efficient estimation regression parameters within a Bayesian framework, without the need to employ computationally intensive Markov Chain Monte Carlo algorithms. Default priors were assigned for all fixed-effect parameters as recommended by^63^, which are approximations of non-informative priors designed to have little influence on the posterior distribution. Specifically, the default prior for fixed effects is specified by a Gaussian distribution with mean of 0 and a precision parameter τ = 0.001. The prior for region hyperparameter is a log-gamma distribution with shape parameter of a = 1 and scale parameter b = 0.00005. The spatial random effect component is defined in terms of two hyperparameters, κ and τ which are related with the range and scale of the spatial effect. Their priors are centred values such that the range is about 20% of the diameter of the region and the variance is equal to 1, see^62^.

Prior to analyses, we scaled all predictor variables (i.e. the five NDVI variables, PA and year), to their overall mean. We also scaled and centered the response variable to aid convergence, because the original body condition values were in the order of thousands. We reiterate here that while body measurements were taken from nestlings of different age, there is no need to account for age in the models because age was already explicitly taken into account in the response when calculating the scaled mass index (see above).

We ran separate models for each of the two vulture species. There was a relatively high degree of collinearity (r > 0.7) among the NDVI variables and, for the white-backed vulture only, between NDVI variables and PA coverage (Fig. S1 in Supplementary Methods). Thus, each predictor was tested in turn in a separate model where year was always included as continuous (variable *time*, see above), and with a random structure as detailed above. We also ran a separate model to test for possible interactive effects between each of the five NDVI variables and PA coverage for the lappet-faced vulture data where collinearity between NDVI and PA coverage was low (i.e. r < 0.7; Fig. S1 in Supplementary Material). This was done because the magnitude of impact of changes in NDVI on nestling body condition may vary according to the PA coverage. This interaction was not tested for the white-backed vulture data due to the strong collinearity (r > 0.7) between PA coverage and NDVI in these data (Fig. S1 in Supplementary Material). Residual spatial autocorrelation was assessed by means of investigating correlograms, but no signs of spatial autocorrelation were found (see Fig. S4 in Supplementary Material). Analyses were performed in R software (version 3.4.0^64^). The cover of PA around each nest was extracted using ArcGIS 10.1.0 software (ESRI).

## Electronic supplementary material

Supplementary Material

## Data Availability

The dataset generated and analysed during the current study is available in the figshare repository, 10.6084/m9.figshare.6876752.

## References

[1] Pacifici M (2017). Species’ traits influenced their response to recent climate change. Nature Clim. Change.

[2] Scheffers, B. R. *et al*. The broad footprint of climate change from genes to biomes to people. *Science* **354**, 10.1126/science.aaf7671 (2016). 10.1126/science.aaf7671 27846577

[3] IPCC. Climate change 2013: the physical science basis. Contribution of Working Group I to the fifth assessment report of the Intergovernmental Panel on Climate Change. 1535 (Cambridge University Press, Cambridge, United Kingdom and New York, NY, USA, 2013).

[4] Jetz W, Wilcove DS, Dobson AP (2007). Projected Impacts of Climate and Land-Use Change on the Global Diversity of Birds. Plos Biology.

[5] Thuiller W (2006). Vulnerability of African mammals to anthropogenic climate change under conservative land transformation assumptions. Global Change Biology.

[6] Dinerstein E (2017). An ecoregion-based approach to protecting half the terrestrial realm. BioScience.

[7] Jetz W (2014). Global Distribution and Conservation of Evolutionary Distinctness in Birds. Current Biology.

[8] Naidoo R (2016). Complementary benefits of tourism and hunting to communal conservancies in Namibia. Conserv. Biol..

[9] Van Asselen S, Verburg PH (2013). Land cover change or land-use intensification: simulating land system change with a global-scale land change model. Global Change Biology.

[10] Cunningham SJ, Madden CF, Barnard P, Amar A (2016). Electric crows: powerlines, climate change and the emergence of a native invader. Diversity and Distributions.

[11] Cunningham SJ, Martin RO, Hojem CL, Hockey PAR (2013). Temperatures in Excess of Critical Thresholds Threaten Nestling Growth and Survival in A Rapidly-Warming Arid Savanna: A Study of Common Fiscals. Plos One.

[12] Pearce-Higgins, J. W. & Green, R. E. *Birds and climate change - Impacts and conservation responses*. 1–467 (Cambridge University Press, 2014).

[13] The Heinz Center. Climate-change vulnerability and adaptation strategies for Africa’s charismatic megafauna. 56 (Washington, DC, 2012).

[14] Mundy, P. J., Butchart, D., Ledger, J. A. & Piper, S. E. *The Vultures of Africa*. (Acorn books and Russel Friedman books, 1992).

[15] Buechley ER, Şekercioğlu ÇH (2016). The avian scavenger crisis: Looming extinctions, trophic cascades, and loss of critical ecosystem functions. Biol. Conserv..

[16] Ogada DL, Torchin ME, Kinnaird MF, Ezenwa VO (2012). Effects of vulture declines on facultative scavengers and potential implications for mammalian disease transmission. Conserv. Biol..

[17] Newton, I. *Population limitation in birds*. (Academic Press, 1998).

[18] Dodge, S. *et al*. Environmental drivers of variability in the movement ecology of turkey vultures (Cathartes aura) in North and South America. *Philosophical Transactions of the Royal Society B: Biological Sciences* **369**, 10.1098/rstb.2013.0195 (2014). 10.1098/rstb.2013.0195 PMC3983930 24733950

[19] Phipps, W. L., Willis, S. G., Wolter, K. & Naidoo, V. Foraging Ranges of Immature African White-Backed Vultures (Gyps africanus) and Their Use of Protected Areas in Southern Africa. *Plos One* **8**, 10.1371/journal.pone.0052813 (2013). 10.1371/journal.pone.0052813 PMC3559650 23382824

[20] Spiegel O, Getz WM, Nathan R (2013). Factors Influencing Foraging Search Efficiency: Why Do Scarce Lappet-Faced Vultures Outperform Ubiquitous White-Backed Vultures?. Am. Nat..

[21] Spiegel O, Harel R, Getz WM, Nathan R (2013). Mixed strategies of griffon vultures’ (Gyps fulvus) response to food deprivation lead to a hump-shaped movement pattern. Movement Ecology.

[22] Kendall, C. J., Virani, M. Z., Hopcraft, J. G. C., Bildstein, K. L. & Rubenstein, D. I. African Vultures Don’t Follow Migratory Herds: Scavenger Habitat Use Is Not Mediated by Prey Abundance. *Plos One* **9**, 10.1371/journal.pone.0083470 (2014). 10.1371/journal.pone.0083470 PMC3885425 24421887

[23] Kendall C, Virani MZ, Kirui P, Thomsett S, Githiru M (2012). Mechanisms of Coexistence in Vultures: Understanding the Patterns of Vulture Abundance at Carcasses in Masai Mara National Reserve, Kenya. The Condor.

[24] Kane A, Kendall CJ (2017). Understanding how mammalian scavengers use information from avian scavengers: cue from above. Journal of Animal Ecology.

[25] Santangeli A, Hakkarainen H, Laaksonen T, Korpimaki E (2012). Home range size is determined by habitat composition but feeding rate by food availability in male Tengmalm’s owls. Animal Behaviour.

[26] Virani MZ, Monadjem A, Thomsett S, Kendall C (2012). Seasonal variation in breeding Ruppell’s Vultures Gyps rueppellii at Kwenia, southern Kenya and implications for conservation. Bird Conserv. Int..

[27] Grande JM (2009). Survival in a long-lived territorial migrant: effects of life-history traits and ecological conditions in wintering and breeding areas. Oikos.

[28] Spiegel O (2015). Moving beyond Curve Fitting: Using Complementary Data to Assess Alternative Explanations for Long Movements of Three Vulture Species. The American Naturalist.

[29] Phipps, W. L., Wolter, K., Michael, M. D., MacTavish, L. M. & Yarnell, R. W. Do Power Lines and Protected Areas Present a Catch-22 Situation for Cape Vultures (Gyps coprotheres)? *Plos One* **8**, 10.1371/journal.pone.0076794 (2013). 10.1371/journal.pone.0076794 PMC3793913 24137496

[30] Oliver TH, Morecroft MD (2014). Interactions between climate change and land use change on biodiversity: attribution problems, risks, and opportunities. Wiley Interdisciplinary Reviews: Climate Change.

[31] Lindsey PA (2013). Benefits of wildlife-based land uses on private lands in Namibia and limitations affecting their development. Oryx.

[32] Murn C, Anderson MD (2008). Activity patterns of African White-backed Vultures Gyps africanus in relation to different land-use practices and food availability. Ostrich.

[33] Buij R, Folkertsma I, Kortekaas K, De Iongh HH, Komdeur J (2013). Effects of land-use change and rainfall in Sudano-Sahelian West Africa on the diet and nestling growth rates of an avian predator. Ibis.

[34] Cruz-McDonnell KK, Wolf BO (2016). Rapid warming and drought negatively impact population size and reproductive dynamics of an avian predator in the arid southwest. Global Change Biology.

[35] Arbeiter S, Schulze M, Tamm P, Hahn S (2016). Strong cascading effect of weather conditions on prey availability and annual breeding performance in European bee-eaters Merops apiaster. Journal of Ornithology.

[36] Ogutu JO, Piepho HP, Dublin HT, Bhola N, Reid RS (2008). Rainfall influences on ungulate population abundance in the Mara-Serengeti ecosystem. Journal of Animal Ecology.

[37] Lindeque PM, Turnbull PCB (1994). Ecology and epidemiology of anthrax in the Etosha National Park, Namibia. Onderstepoort journal of veterinary research.

[38] Shorrocks, B. & Bates, W. *The biology of African savannahs*. 2nd Edition edn, (Oxford University press, 2015).

[39] Thuiller W (2006). Endemic species and ecosystem sensitivity to climate change in Namibia. Global Change Biology.

[40] Hopcraft JGC, Olff H, Sinclair ARE (2010). Herbivores, resources and risks: alternating regulation along primary environmental gradients in savannas. Trends in Ecology & Evolution.

[41] Tucker MA (2018). Moving in the Anthropocene: Global reductions in terrestrial mammalian movements. Science.

[42] Phipps WL (2017). Due South: A first assessment of the potential impacts of climate change on Cape vulture occurrence. Biol. Conserv..

[43] Santangeli A, Arkumarev V, Komen L, Bridgeford P, Kolberg H (2017). Unearthing poison use and consequent anecdotal vulture mortalities in Namibia’s commercial farmland – implications for conservation. Ostrich.

[44] Santangeli A, Arkumarev V, Rust N, Girardello M (2016). Understanding, quantifying and mapping the use of poison by commercial farmers in Namibia – Implications for scavengers’ conservation and ecosystem health. Biol. Conserv..

[45] Garbett R (2018). Association between hunting and elevated blood lead levels in the critically endangered African white-backed vulture Gyps africanus. Science of The Total Environment.

[46] Monadjem A, Botha A, Murn C (2013). Survival of the African white-backed vulture Gyps africanus in north-eastern South Africa. Afr. J. Ecol..

[47] Monadjem A, Wolter K, Neser W, Kane A (2014). Effect of rehabilitation on survival rates of endangered Cape vultures. Anim. Conserv..

[48] Martin TE (1987). Food as a limit on breeding birds - A life-history perspective. Annu. Rev. Ecol. Syst..

[49] Schwagmeyer PL, Mock DW (2008). Parental provisioning and offspring fitness: size matters. Animal Behaviour.

[50] O’Connor TG, Puttick JR, Hoffman MT (2014). Bush encroachment in southern Africa: changes and causes. African Journal of Range & Forage Science.

[51] Schultz, P. *Does bush encroachment impact foraging success of the critically endangered namibian population of the cape vulture Gyps coprotheres?* Master thesis, University of Cape Town (2007).

[52] Ogada D (2016). Another Continental Vulture Crisis: Africa’s Vultures Collapsing toward Extinction. Conservation Letters.

[53] Hipkiss T, Hörnfeldt B (2004). High interannual variation in the hatching sex ratio of Tengmalm’s owl broods during a vole cycle. Population Ecology.

[54] Mendelsohn, J., Jarvis, A., Roberts, C. & Robertson, T. *Atlas of Namibia: A portrait of the land and its people*. (David Philip Publishers, 2002).

[55] Simmons, R. E., Brown, C. J. & Kemper, J. *Birds to watch in Namibia: red*, *rare and endemic species*. 1–319 (Ministry of Environment and Tourism and Namibia Nature Foundation, 2015).

[56] Peig J, Green AJ (2009). New perspectives for estimating body condition from mass/length data: the scaled mass index as an alternative method. Oikos.

[57] Resano-Mayor J (2016). The influence of diet on nestling body condition of an apex predator: a multi-biomarker approach. Journal of Comparative Physiology B.

[58] Pettorelli N (2005). Using the satellite-derived NDVI to assess ecological responses to environmental change. Trends in Ecology & Evolution.

[59] Rasmussen HB, Wittemyer G, Douglas-Hamilton I (2006). Predicting time-specific changes in demographic processes using remote-sensing data. J. Appl. Ecol..

[60] Wato YA (2016). Prolonged drought results in starvation of African elephant (Loxodonta africana). Biol. Conserv..

[61] NASA LP DAAC. MODIS/Terra+ Aqua Vegetation Indices 16-Day L3 Global 250m (MOD13Q1). USGS Earth Resources Observation and Science (EROS) Center, Sioux Falls, South Dakota, https://lpdaac.usgs.gov, accessed January 31, 2017, at, 10.5067/MODIS/MOD13Q1.005 (2017).

[62] Lindgren F, Rue H, Lindström J (2011). An explicit link between Gaussian fields and Gaussian Markov random fields: the stochastic partial differential equation approach. Journal of the Royal Statistical Society: Series B (Statistical Methodology).

[63] Held, L., Schrödle, B. & Rue, H. In *Statistical Modelling and Regression Structures: Festschrift in Honour of* Ludwig Fahrmeir (eds Thomas Kneib & Gerhard Tutz) 91–110 (Physica-Verlag HD, 2010).

[64] R Core Development Team. *R: A language and environment for statistical computing*. *Version* 3.4.0. (Available from, https://www.r-project.org/ 2016).

