# Supplementary material for: Synergistic effect of land-use and vegetation greenness on vulture nestling body condition in arid ecosystems
Source: Sci Rep. 2018 Aug 29;8:13027. doi: 10.1038/s41598-018-31344-2 (PMC6115393; doi:10.1038/s41598-018-31344-2)
Supplement: Supplementary file 1 — Supplementary Material [file 41598_2018_31344_MOESM1_ESM.pdf]

Synergistic effect of land-use and vegetation greenness on vulture nestling body condition in arid ecosystems

Authors: *Andrea Santangeli*[1, *], *Orr Spiegel*[2], *Peter Bridgeford*[3], *Marco Girardello*[4]

Affiliations:

[1] The Helsinki Lab of Ornithology, Finnish Museum of Natural History, University of Helsinki, Finland

[2] School of Zoology, Faculty of Life Sciences, Tel Aviv University, Tel Aviv 69978, Israel

[3] Vultures Namibia, Walvis Bay, Namibia

[4] cE3c - Centre for Ecology, Evolution and Environmental Changes/Azorean Biodiversity Group and Universidade. dos Açores – Depto de Ciências e Engenharia do Ambiente, PT-9700-042, Angra do Heroísmo, Açores, Portugal.

*Correspondence to: andrea.santangeli@helsinki.fi; tel: +358 504484443; fax: +358 2941 57694

Supplementary Figures

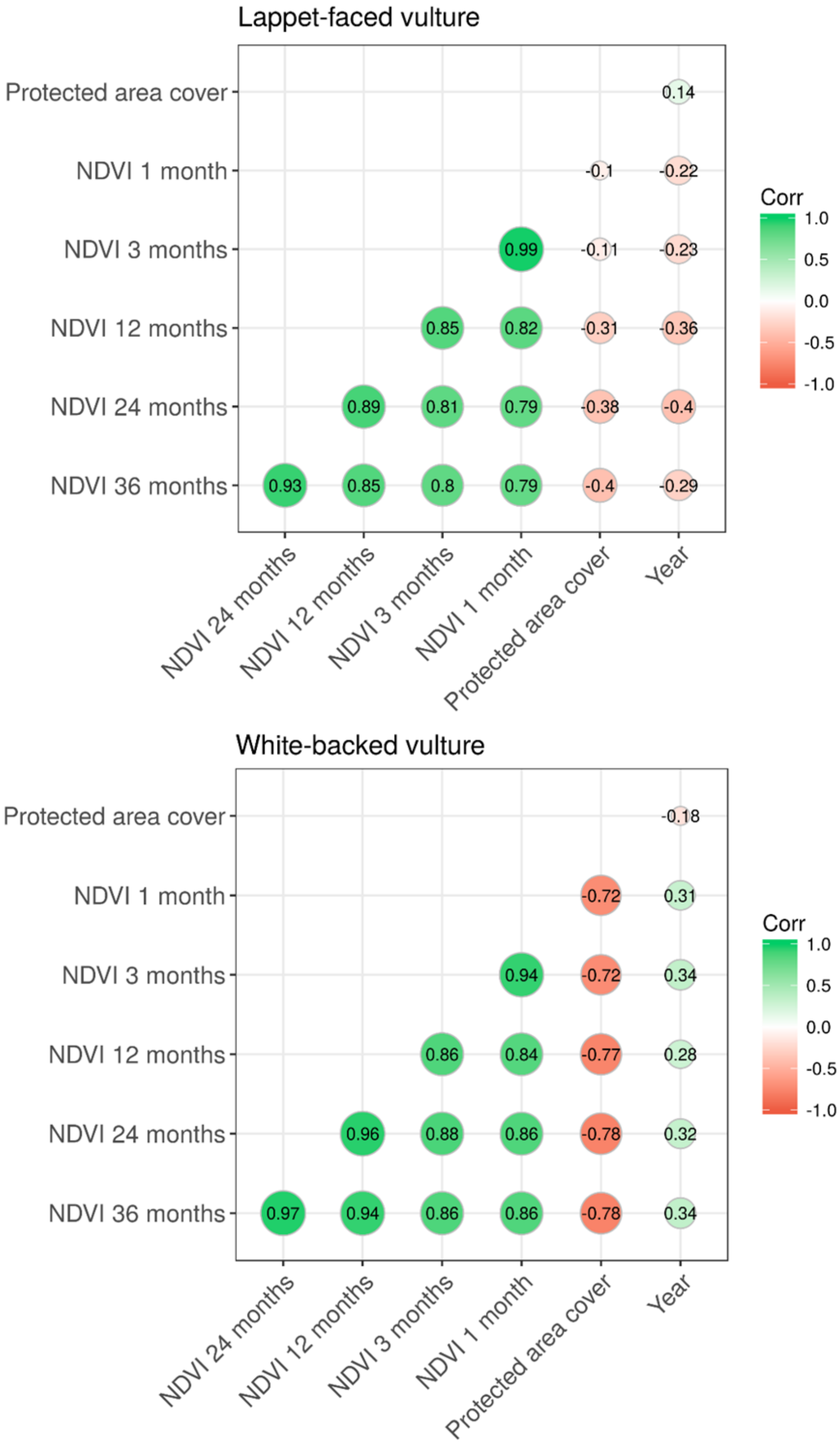

Figure S1. Correlation between explanatory variables calculated using the spearman rho correlation coefficient separately with data from the lappet-faced and white-backed vulture nestling. The size of the circle is proportional to the value of the correlation coefficient (shown within each circle), whereas the direction of the correlation is depicted by the colour pattern, from red to green (see scale).

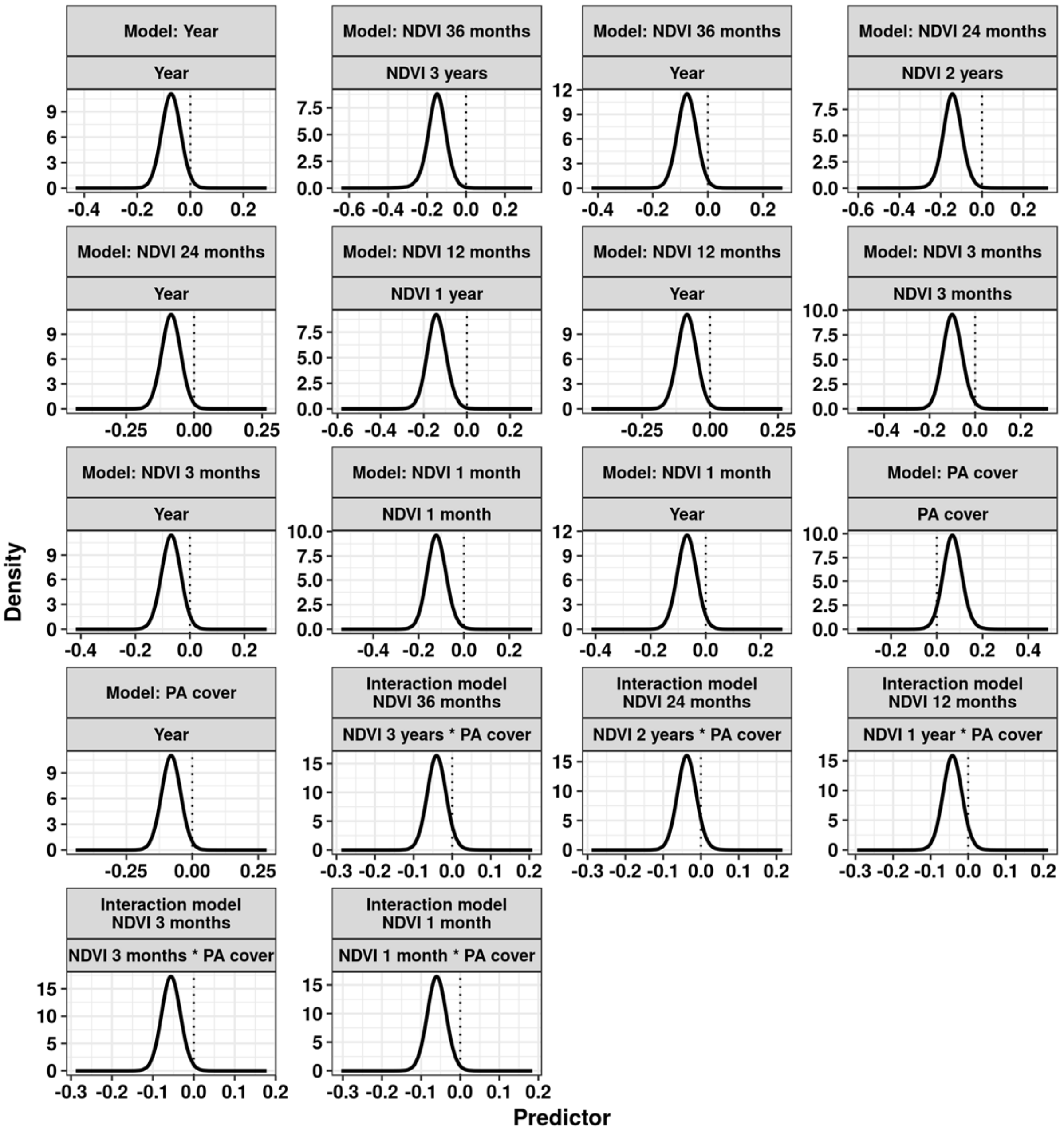

Fig S2. Posterior (marginal) distributions for the fixed effects of the models fitted to the lappet-facet vulture body condition dataset.

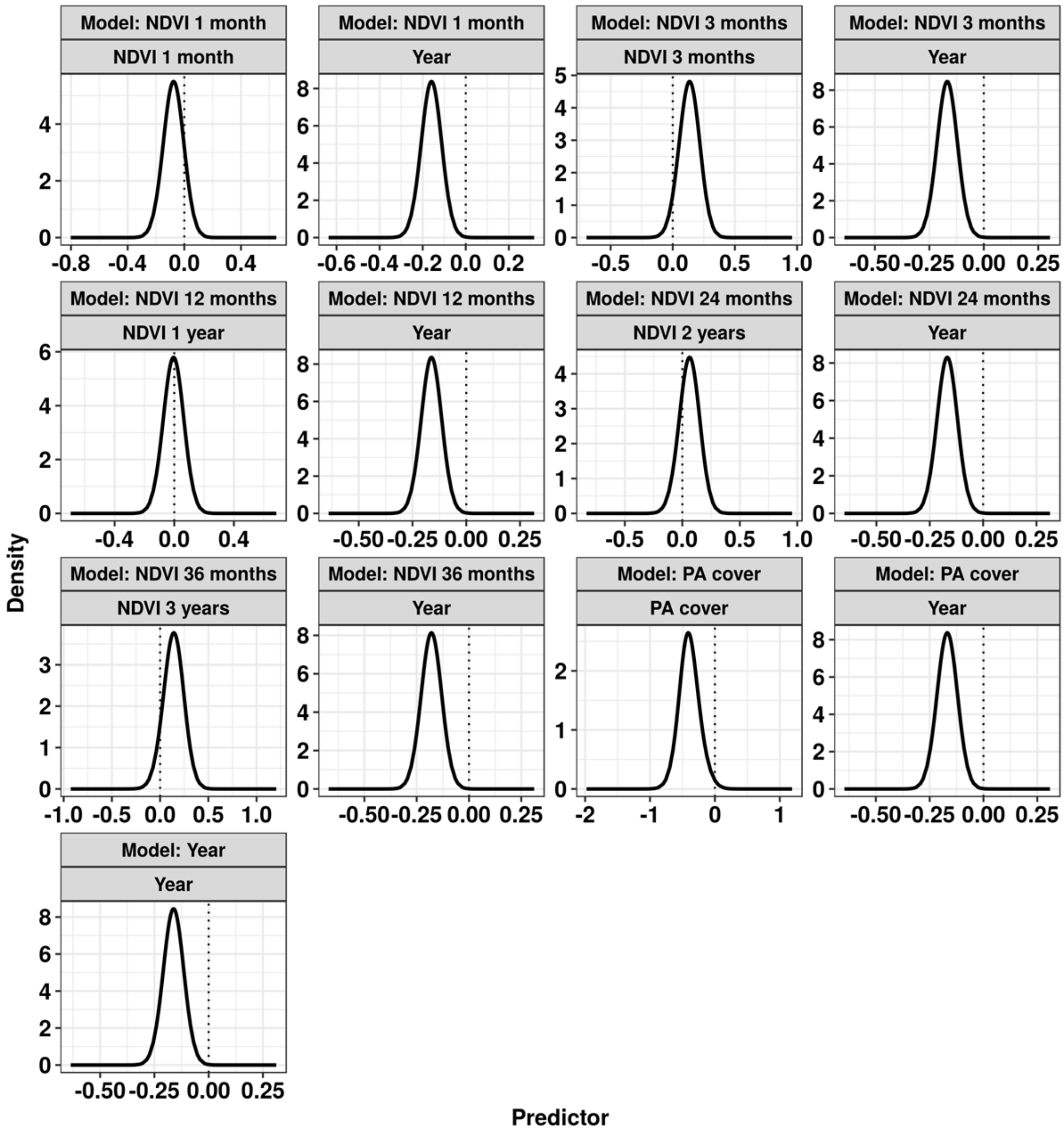

Fig S3. Posterior (marginal) distributions for the fixed effects of the models fitted to the white-backed vulture body condition dataset.

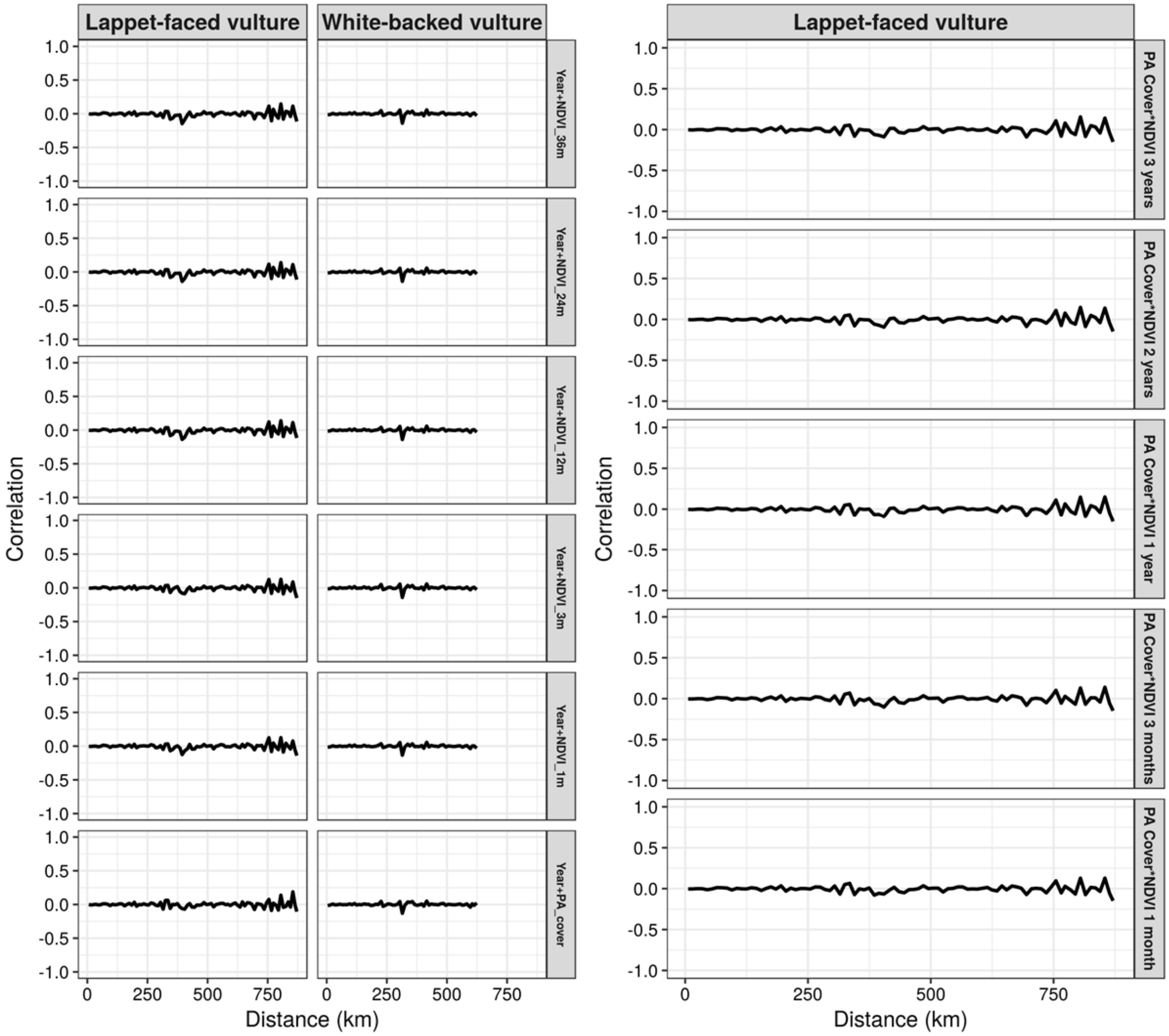

Figure S4. The spatial autocorrelation by distance as assessed by Morans' I autocorrelation function based on the residuals from each model presented in the main manuscript (Table 1). The relevant species for which each panel applies is stated on top of the figures, and to the right is shown the specific model based on the predictors considered. Note here "Year" stands for Time as in Table 1 as it was considered as a continuous variable in the fixed part of the models. Values aligning to the Y = 0 line represent total absence of any spatial autocorrelation, which is almost never the case for these types of data.
